# Aldo-keto reductase family member C3 (AKR1C3) promotes hepatocellular carcinoma cell growth by producing prostaglandin F2α

**DOI:** 10.32604/or.2023.030975

**Published:** 2023-11-15

**Authors:** KUO-SHYANG JENG, PO-YU CHENG, YUEH-HSIEN LIN, PO-CHUN LIU, PING-HUI TSENG, YU-CHAO WANG, CHIUNG-FANG CHANG, CHUEN-MIIN LEU

**Affiliations:** 1 Division of General Surgery, Far Eastern Memorial Hospital, New Taipei City, 22060, Taiwan; 2 Institute of Microbiology and Immunology, National Yang Ming Chiao Tung University, Taipei City, 11221, Taiwan; 3 Institute of Biochemistry and Molecular Biology, National Yang Ming Chiao Tung University, Taipei City, 11221, Taiwan; 4 Institute of Biomedical Informatics, National Yang Ming Chiao Tung University, Taipei City, 11221, Taiwan; 5 Department of Medical Research, Far Eastern Memorial Hospital, New Taipei City, 22060, Taiwan

**Keywords:** Hepatocellular carcinoma, Aldo-keto reductase family member C3, Prostaglandin F2 alpha, Prostaglandin F receptor

## Abstract

Hepatocellular carcinoma (HCC) is a leading cause of death worldwide. Current therapies are effective for HCC patients with early disease, but many patients suffer recurrence after surgery and have a poor response to chemotherapy. Therefore, new therapeutic targets are needed. We analyzed gene expression profiles between HCC tissues and normal adjacent tissues from public databases and found that the expression of genes involved in lipid metabolism was significantly different. The analysis showed that AKR1C3 was upregulated in tumors, and high AKR1C3 expression was associated with a poorer prognosis in HCC patients. *In vitro*, assays demonstrated that the knockdown of AKR1C3 or the addition of the AKR1C3 inhibitor indomethacin suppressed the growth and colony formation of HCC cell lines. Knockdown of AKR1C3 in Huh7 cells reduced tumor growth *in vivo*. To explore the mechanism, we performed pathway enrichment analysis, and the results linked the expression of AKR1C3 with prostaglandin F2 alpha (PGF2α) downstream target genes. Suppression of AKR1C3 activity reduced the production of PGF2α, and supplementation with PGF2α restored the growth of indomethacin-treated Huh7 cells. Knockdown of the PGF receptor (PTGFR) and treatment with a PTGFR inhibitor significantly reduced HCC growth. We showed that indomethacin potentiated the sensitivity of Huh7 cells to sorafenib. In summary, our results indicate that AKR1C3 upregulation may promote HCC growth by promoting the production of PGF2α, and suppression of PTGFR limited HCC growth. Therefore, targeting the AKR1C3-PGF2α-PTGFR axis may be a new strategy for the treatment of HCC.

## Introduction

Hepatocellular carcinoma (HCC) is one of the deadliest cancers with a high incidence rate, especially in Asian countries. Major risk factors for HCC include chronic liver inflammation caused by persistent infection with hepatitis B or hepatitis C virus, obesity, and alcohol abuse [1–3]. Local ablation, surgical resection, and liver transplantation are effective treatments for patients with early disease, but many HCC patients experience recurrence after surgery and have a poor response to chemotherapy. Sorafenib was the first drug approved for advanced-stage primary HCC, but the response rate is low [4]. To date, sorafenib and lenvatinib are the only first-line drugs prescribed for unresectable HCC; however, these two drugs have a high rate of treatment-related adverse effects or toxicities, which may lead to dose reduction or treatment interruption [5]. The combination of atezolizumab and bevacizumab in clinical trials showed better overall and progression-free survival outcomes than sorafenib in patients with unresectable HCC [6]. Until more effective therapy is approved, there is an unmet need for new therapeutic targets in the treatment of HCC patients.

Aldo-keto reductase family 1 member C3 (AKR1C3) is a versatile enzyme that catalyzes the biosynthesis of testosterone and estradiol [7–9]. AKR1C3 is also able to convert prostaglandin H2 and D2 into prostaglandin F2 alpha (PGF2α) and 9α,11β-PGF2α, respectively [10]. The upregulation of AKR1C3 is associated with progression, aggressiveness, and drug resistance in prostate cancer [11–14]. In breast cancer, AKR1C3 mediates doxorubicin resistance by activating AKT via PTEN loss [15]. An increase in AKR1C3 expression in HCC patients has been reported and it is correlated with a lower overall survival rate [16]. Another study demonstrated that AKR1C3 induced the activation of TRAF6 and downstream NF-κB to stimulate proinflammatory cytokine IL-6 production, which promotes the proliferation and invasion of HCC cells [17]. Although their findings revealed a critical role of AKR1C3 in HCC development, whether AKR1C3 controls the growth of HCC via other mechanisms is not clear.

Prostaglandins (PGs) are biologically active lipid mediators that participate in diverse physiological and pathological conditions. Each member in the PG family is synthesized from arachidonic acid first by cyclooxygenase followed by a specific synthase. A large body of evidence identified the importance of prostaglandin E (PGE) and its receptors in the development, progression, and metastasis of many cancers, including HCC [18,19]. However, the roles of PGF and its receptor PTGFR (also called FP) in cancer development are less clear. PGF2α and 9α,11β-PGF2α bind the same PTGFR with similar affinity and exert similar biological functions. Under normal physiological conditions, PGF is produced by the uterus when stimulated with oxytocin. The PGF2α-PTGFR pathway promotes proliferation, migration, and angiogenesis in endometrial carcinoma [20–22]. PGF2α also stimulates the migration and invasion of colorectal carcinoma cells and the survival of breast cancer cells [23,24]. However, whether the PGF2α-PTGFR pathway is involved in HCC development is unknown.

To search for new therapeutic targets, we analyzed differentially expressed genes between HCC and normal adjacent liver tissues from public databases. Aside from lipogenesis pathway genes, whether other genes in lipid metabolism affect the tumorigenesis of HCC is not clear. Our analysis showed that eight genes in lipid metabolism were significantly upregulated in HCC tissues, and high expression of these genes was associated with a poorer prognosis. Whether AKR1C3 and its product PGF2α regulate the growth of human HCC remains to be clarified. Here we studied whether AKR1C3 promotes HCC cell growth by producing PGF2α.

## Materials and Methods

### Data collection and process

Four datasets containing the gene expression profiles of HCC tumors and adjacent normal tissues (GSE39791, GSE57957, GSE76427, and GSE84598, shown in Table 1) were downloaded from the Gene Expression Omnibus (GEO) database. The raw data first underwent quantile normalization using the “limma” package [25]. The data were transformed using log2, and unknown genes were removed using the “stringr” package [26]. Significantly differentially expressed genes were defined as genes with a fold-change ≥ 1.5, *p* < 0.05, paired sample *t-*test. The gene expression profiles and overall survival of a total of 365 patients in The Liver Hepatocellular Carcinoma (LIHC) dataset in The Cancer Genome Atlas (TCGA) were downloaded, and there were a total of 50 patients with paired tumor and normal tissue expression profiles. For the survival analysis, the TCGA dataset was divided into an AKR1C3 high-expression group (n = 182) and a low-expression group (n = 183) based on the median AKR1C3 level. The log-rank test was used to estimate differences in survival between the high- and low-expression groups.

**TABLE 1:** Information of HCC patients from the Gene Expression Omnibus

Series accession	Tissue	Tumor & adjacent normal tissues	Platform	Source
GSE39791	Liver	72 pairs	GPL10558 (48017 probe sets)	Korean
GSE57957		36 pairs		Asian
GSE76427		52 pairs		Singapore
GSE84598		22 pairs		Germany

### Clinical specimens

To confirm the upregulation of AKR1C3 in the open databases, a total of 21 HCC tumor tissues and adjacent non-tumor tissues were collected from Far Eastern Memorial Hospital (New Taipei City, Taiwan). The clinical samples were kept at −80°C until use. The diagnosis of HCC was confirmed histologically in all cases. The demographic characteristics of the HCC patients are listed in Suppl. Table S1. The institutional ethics committees of Far Eastern Memorial Hospital (IRB protocol No. 110004F) and National Yang Ming Chiao Tung University (YM109128E) approved this study, and informed consent was obtained from all patients.

### Quantitative RT-PCR

Total RNA was extracted from tissues or cell lines using TRIzol reagent (Invitrogen, MA, USA). RNA was reverse transcribed using a SuperScript III First-Strand Synthesis System (Invitrogen). The following primers were used for q-PCR: AKR1C3, forward, 5-GTTCCGCCATATAGATTCTGC-3′, reverse, 5′-CTCTGGTCGATGAAAAGTGG3′; PTGFR, forward, 5′-CATCAATGGAGCCATAGCAG-3′, reverse, 5′- TGACTCCAATACACCGCTCA-3′. Quantitative RT-PCR was performed using the SYBR Green Master mix kit (Thermo, MA, USA). GAPDH was used as a normalization control.

### Western blotting

Tissues or cell lines were lysed using RIPA lysis buffer containing protease inhibitors. Western blotting was performed as described [27]. Anti-AKR1C3 (GeneTex, CA, USA), anti-GAPDH (GeneTex), and anti-α-tubulin (Abcam, Cambridge, UK).

### Cell culture

Huh-7, Hep3B, PLC/PRF/5, and 293T cells were cultured in Dulbecco’s modified Eagle’s medium (DMEM) containing 10% fetal calf serum (Gibco, CA, USA) in humidified 5% CO_2_ at 37°C. Huh7 cells were obtained from the Japanese Collection of Research Bioresources (Osaka, Japan). Hep3B cells were from ATCC. PLC/PRF/5 cells were from Bioresource Collection and Research Center (Hsinchu, Taiwan). An AKR1C3 overexpression lentiviral vector was constructed by inserting PCR-amplified human AKR1C3 cDNA into pLAS3W.PeGFP.I2.puro. The shRNA knockdown lentiviral vectors for AKR1C3 and PTGFR were purchased from the RNAi Core facility in Academia Sinica (Taipei City, Taiwan). Overexpression or shRNA knockdown lentiviral vectors specific to AKR1C3 or PTGFR were co-transfected with packing vectors into 293T cells, and the viruses in the supernatant were used to transduce HCC cell lines. One day after transduction, these cell lines were selected using 1 μg/mL puromycin for a least 1 week to obtain stable cell lines.

### Cell growth

Various numbers of different HCC cells were seeded into 24-well plates. Different inhibitors (AKR1C3, PTGFR, and sorafenib) were added on the second day, and the cell number was calculated using trypan blue. Indomethacin was purchased from Sigma-Aldrich (MO, USA). AL-8810, PGF2α, and sorafenib were purchased from Cayman Chemical (MI, USA).

### Colony forming assay

Vector control and overexpressing/knockdown stable lines of Huh7, Hep3B, and PLC/PRF/5 were seeded at a low density (300–500 cells/dish) in 6-well plates and cultured for 12 days. The medium was replaced every 4 days. After washing with PBS, colonies were stained with 0.5% crystal violet in 4% formaldehyde for 15 min. The average number of colonies out of 4 fields in each group was evaluated using ImageJ.

### In vivo tumorigenesis assay

This study has been reviewed and approved by the Institutional Animal Care and Use Committee at National Yang Ming Chiao Tung University (approval No. 1091108). One and a half million vector control or AKR1C3 knockdown Huh7 (shRNA#2) cells were mixed with Matrigel (Corning, NY, USA), and injected subcutaneously into the left or right back of the same nude mouse (CAnN.Cg-Foxn1nu/CrlNarl, purchased from National Laboratory Animal Center, Taipei City, Taiwan). A total of 8 male mice were used. Tumor growth *in vivo* was measured using calipers at week 2 and week 3, and all tumors were harvested after 3 weeks to measure tumor weight.

### Detection of PGF2α

Fifty thousand Huh7 cells were seeded in 24-well plates overnight and washed once with PBS. Serum-free DMEM with or without various concentrations of indomethacin was added, and the conditioned medium was harvested after 72 h. PGF2α in the supernatant was detected using a PGF2α ELISA kit (Enzo Life Sciences, New York, USA) according to the procedures provided by the manufacturer.

### Statistical analysis

**FIGURE 1:**
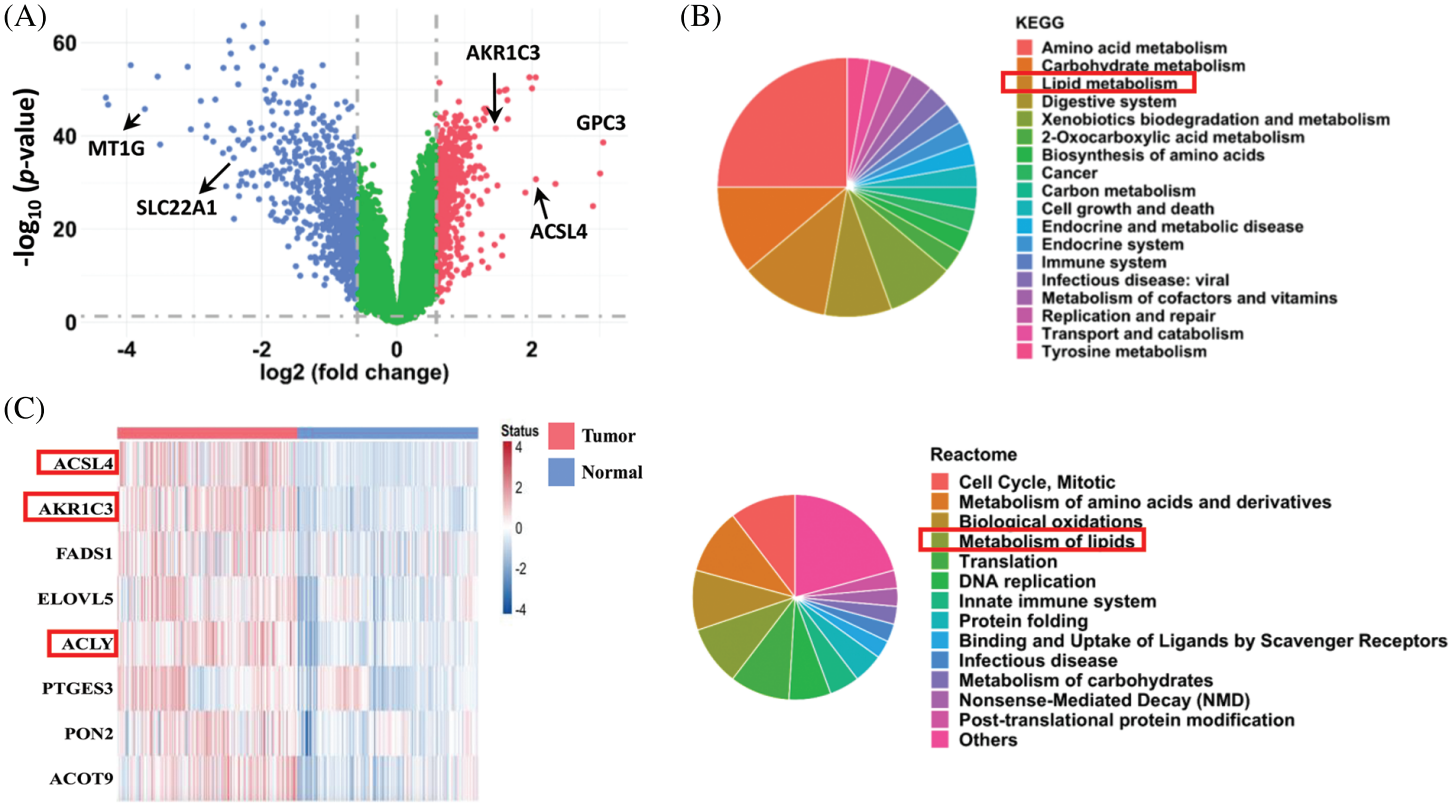
Analysis of differentially expressed genes in human HCC tissues. (A) Differentially expressed genes in human HCC tissues. After quantile normalization, the gene expression levels in the HCC tissues (GSE39791, GSE57957, GSE76427, and GSE84598 from the GEO database) were divided by the levels in normal adjacent tissue to calculate fold changes. Genes with fold-change ≥ 1.5 are shown in red and blue in the plot. A paired sample *t*-test was used to calculate statistical significance. (B) Pathway enrichment analysis (KEGG and Reactome) of the differentially expressed genes in the HCC tissues. (C) Heatmap of upregulated genes in lipid metabolism from the tumor tissues and normal adjacent tissues of HCC patients (from GEO database).

**Figure 2:**
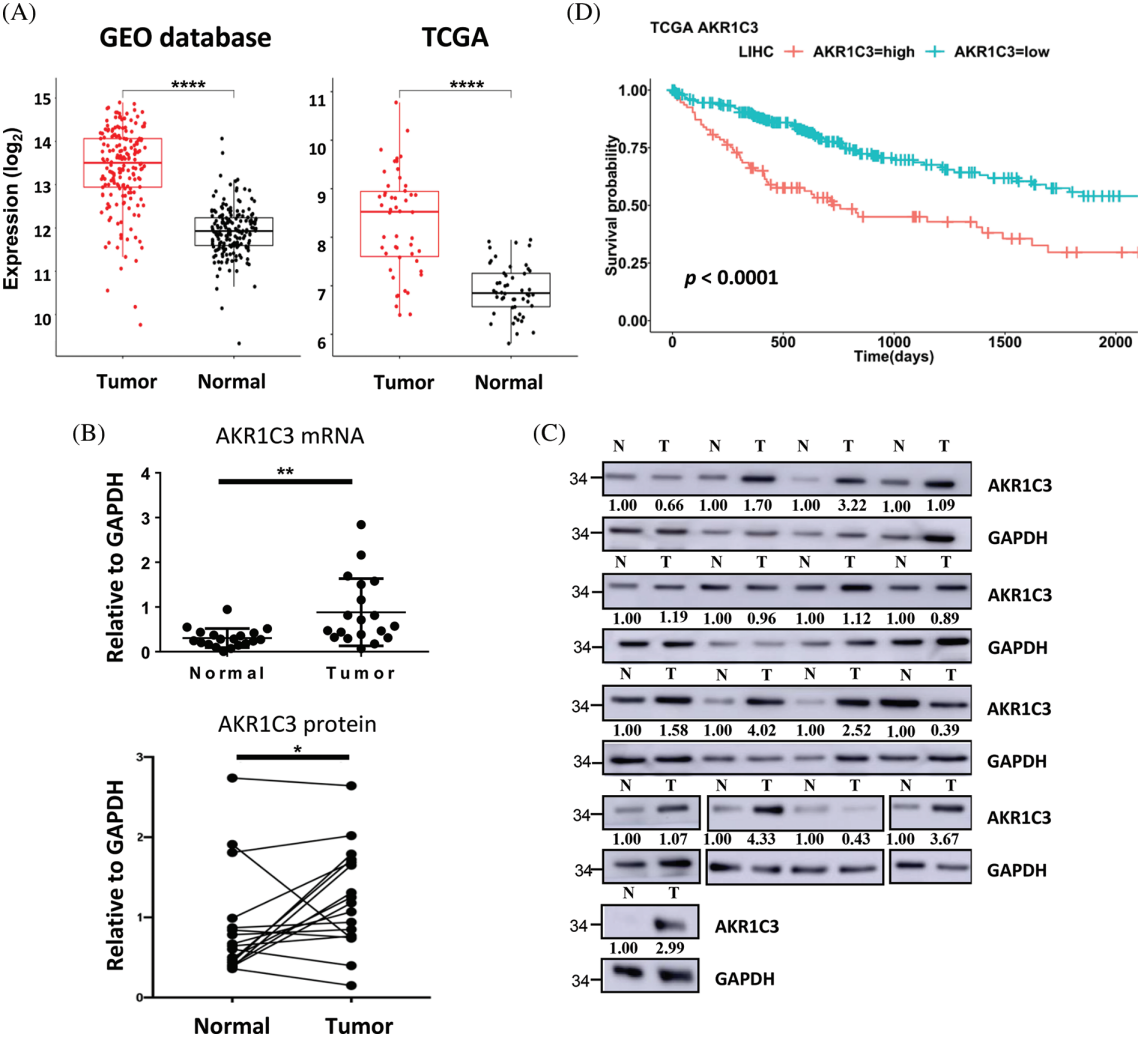
AKR1C3 is upregulated and correlates with poor prognosis in human HCC. (A) The expression of AKR1C3 in paired HCC and normal adjacent liver tissues from the 4 GEO datasets (Table 1, n = 182) or the TCGA dataset (n = 50) is shown. The middle line in each box represents the median of all samples. The upper edge of the rectangular box is the third quartile, and the lower edge of the box is the first quartile. *p < 0.05; **p < 0.01; ****p < 0.0001. RT-qPCR (B) or Western blot analysis (C) of AKR1C3 expression in 19 or 17 pairs of HCC and normal adjacent liver tissues from Far Eastern Memorial Hospital is shown. In B, data are shown as mean ± SD, and the Wilcoxon test was used. (D) Correlation between AKR1C3 expression and overall survival rates in HCC patients from the TCGA database (n = 365). The 365 HCC patients were divided into AKR1C3 high expression (n = 182) and low expression (n = 183) groups. The log-rank test was used.

**Figure 3:**
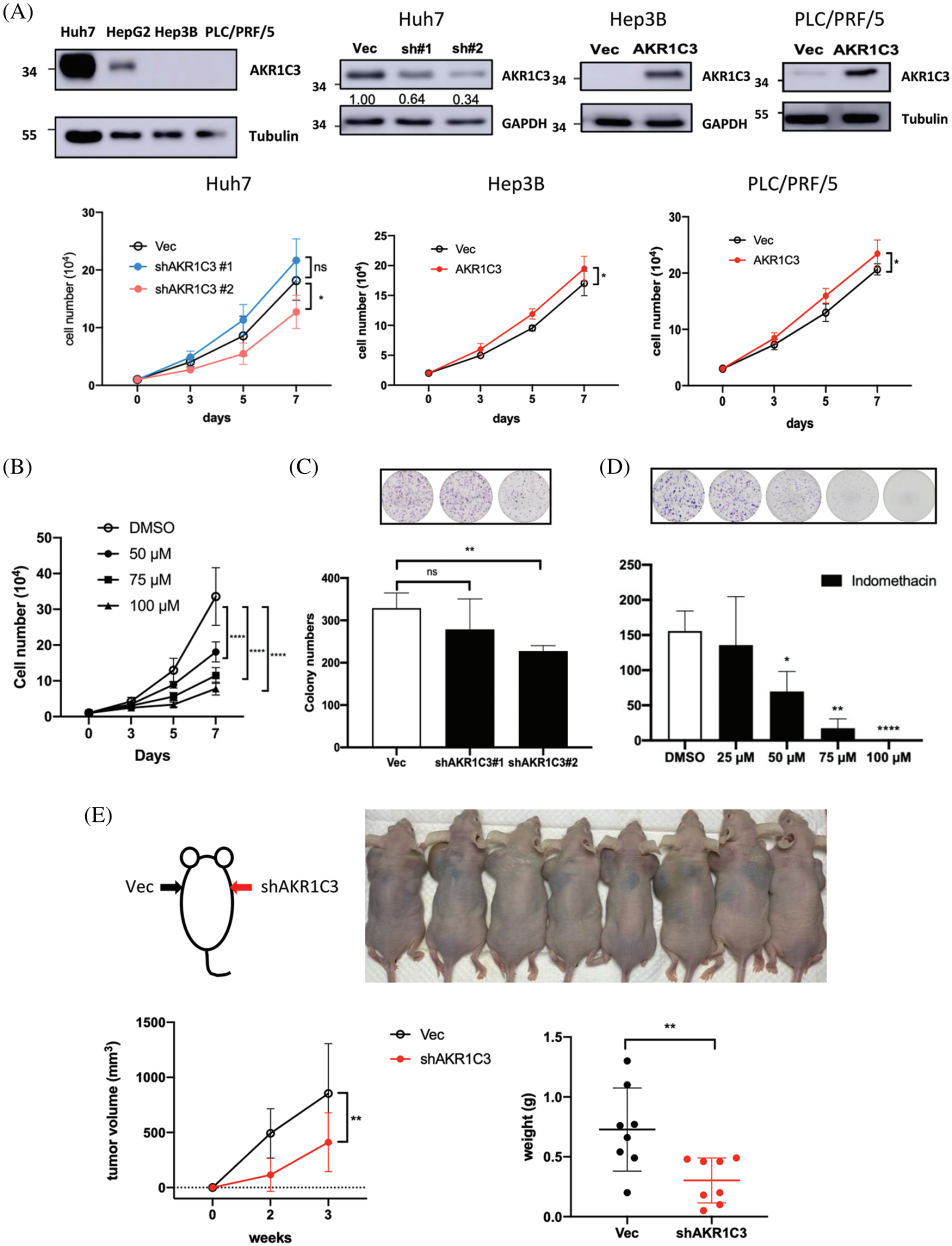
AKR1C3 expression enhances the growth of human HCC cells *in vitro* and *in vivo*. (A) The expression of AKR1C3 in parental HCC cell lines, or stable lines containing vector control (Vec), AKR1C3 knockdown shRNA constructs (sh#1 and sh#2), or overexpression construct (AKR1C3) was examined using Western blotting. A trypan blue exclusion assay was used to evaluate cell growth. The average cell number out of 3~5 independent experiments is shown. (B) The AKR1C3 inhibitor indomethacin suppressed Huh7 cell growth. Knockdown of AKR1C3 (C) or indomethacin (D) suppressed the colony formation of Huh7 cells. Huh7 parental or stably transduced lines were plated on 6-well plates with or without the inhibitor for 12 days. After the cells were washed, fixed, and stained with crystal violet, the colony number was evaluated using ImageJ, and the average colony number out of 5 independent experiments is shown. (E) AKR1C3 promotes human HCC growth *in vivo*. Vector control or AKR1C3 knockdown Huh7 cells (sh#2) were injected subcutaneously into both sides of the backs of nude mice, and the tumor size was measured 2 and 3 weeks after inoculation. The average tumor volume and the tumor weight at week 3 are shown (n = 8). *p < 0.05; **p < 0.01; ****p < 0.0001; All data in this figure are shown as mean ± SD.

**Figure 4:**
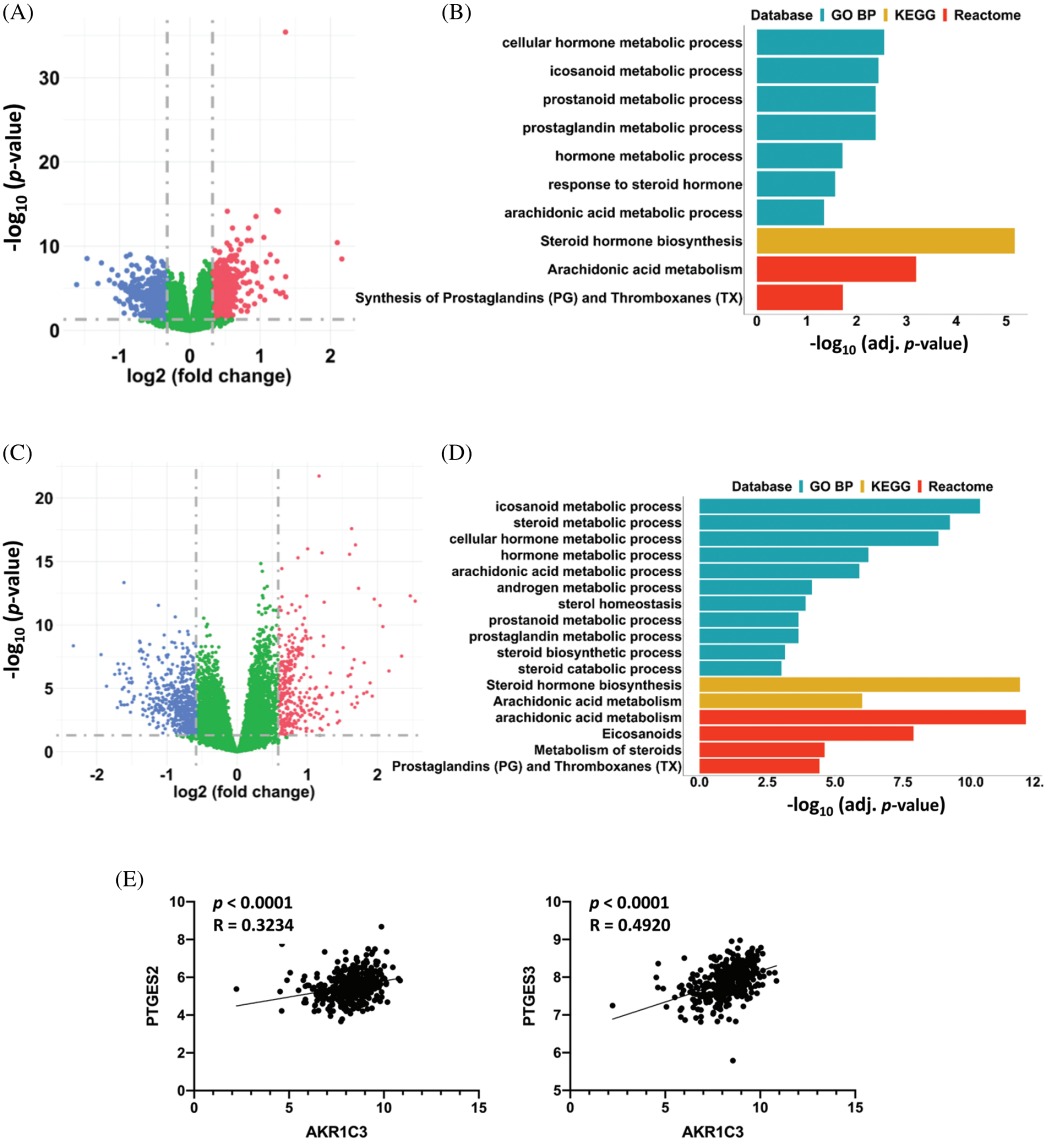
AKR1C3 upregulation associated with changes in prostaglandin metabolism in HCCs. Differentially expressed genes in the 182 human HCC tissues from the GEO datasets (A) or 365 HCC tissues from the TCGA database (C). The HCC tumor tissues were divided into AKR1C3-high and AKR1C3-low groups, and the expression of specific genes in the AKR1C3-high group was divided by the levels in the AKR1C3-low group. A fold-change ≥ 1.5 and *p* < 0.05 were considered significant changes, and a paired sample *t*-test was used. Pathway enrichment analysis (GO BP, KEGG, and Reactome) of the differentially expressed genes between the AKR1C3 high and low groups from the GEO (B) or TCGA database (D). (E) The expression of the PTGFR target genes PTGES2 and PTGES3 was positively associated with AKR1C3 expression. The correlation between AKR1C3 expression and the expression of specific genes from the TCGA database was analyzed using Spearman correlation.

Equal variance Student’s *t-*test (R studio, matrixTests package) was used to analyze differentially expressed genes in Figs. 1A, 4A, and 4C. *p* values less than 0.05 were considered significant. A paired *t*-test (R studio, ggplot package) was used in Fig. 2A. The Wilcoxon test (GraphPad Prism, CA, USA) was used in Fig. 2B. The correlation between AKR1C3 expression and HCC patient survival was analyzed using the log-rank test (R studio, ggplot package). An unpaired *t*-test (Prism) was used in Figs. 3C and 3D. False discovery rates in Figs. 4B and 4D were calculated using the clusterProfiler package in R studio. The correlation between AKR1C3 expression and the expression of specific genes in Fig. 4E was analyzed using Spearman correlation (Prism). The other figures were analyzed using the Mann-Whitney U test (Prism. *p < 0.05; **p < 0.01; ***p < 0.001; ****p < 0.0001).

## Results

### Analysis of differentially expressed genes in human HCC tissues from GEO datasets

To identify novel genes regulating HCC development, we combined 4 datasets (GSE39791, GSE57957, GSE76427, and GSE84598) from the GEO database and compared the gene expression profiles for a total of 182 pairs of tumor and adjacent nontumor tissues from patients with HCC. The analysis showed that 590 genes were significantly upregulated, and 760 genes were downregulated in HCC tumor tissues compared to normal adjacent liver tissues (Fig. 1A, fold-change ≥ 1.5 and *p* < 0.05 were defined as significantly changed). Consistent with previous reports, the expression of *ACSL4* and *GPC3* was increased, and the expression of *MT1G* and *SLC22A1* was decreased in the tumor tissues [28–31]. Based on KEGG and Reactome pathway enrichment analyses, these dysregulated genes were associated with the cell cycle, amino acid metabolism, translation, DNA replication, cell growth, and apoptosis (Fig. 1B). Notably, lipid metabolism was among the top 5 pathways markedly changed in both analyses, and many genes in lipid metabolism were downregulated in the HCC tissues. These results reveal that cell proliferation is very active, and certain metabolic pathways are dysregulated in HCC, which is consistent with previous reports [32–34].

In addition to amino acid and glucose metabolism, a growing body of studies explores the role of lipid metabolism in HCC development. To search for new therapeutic targets, we focused on the upregulated genes in lipid metabolism. Among the 590 upregulated genes, only 8 genes in lipid metabolism were associated with a poorer prognosis, and their expression levels in the 182 pairs of HCC tissues are shown in Fig. 1C. The functions of *ACSL4* and *ACLY* in HCC have been reported [29,35]. Because little was known about the function of AKR1C3 in HCC at the beginning of our research, we focused on it.

### AKR1C3 is significantly upregulated in human HCC tissues

In the 4 GEO datasets, AKR1C3 mRNA expression increased 2.76-fold in the tumor tissues on average (Fig. 2A, GEO database). To confirm this observation, we checked another cohort that contained 50 pairs of HCC tumor and normal tissue expression profiles in the TCGA database. The analysis showed a similar upregulation of AKR1C3 in the HCC tissues (Fig. 2A, TCGA). Furthermore, we examined the mRNA and protein levels of AKR1C3 in the liver tissues collected from the Far Eastern Memorial Hospital and found that AKR1C3 expression was also increased in some if not all, tumors (Figs. 2B and 2C). Collectively, these results confirmed the upregulation of AKR1C3 in HCC tissues from different ethnic groups.

To examine the significance of AKR1C3 upregulation, we investigated the correlation between AKR1C3 expression and the overall survival of HCC patients using a cohort of 365 patients in the TCGA database. These patients were divided into AKR1C3-high (n = 182) and AKR1C3-low groups (n = 183) based on the mRNA level, and the survival rate was analyzed. As shown in Fig. 2D, patients with high AKR1C3 expression had a significantly lower survival rate (*p* < 0.0001). The progression-free survival time was also shorter in the AKR1C3-high HCC patients (Suppl. Fig. S1, *p* < 0.05). We also examined the association of AKR1C3 expression with tumor stage, ethnic group, age, weight, and various risk factors and did not find any correlation (Suppl. Fig. S2). These analyses show that AKR1C3 upregulation is associated with a poor prognosis in HCC patients.

### AKR1C3 expression promotes the growth and colony formation of human HCC cell lines

Because tumor tissues consist of several cell types, the increase in AKR1C3 may come from hepatoma cells, surrounding stromal cells, or leukocytes. To address this question, we measured AKR1C3 expression in human HCC cell lines. Huh7 cells have high AKR1C3 expression, and Hep3B and PLC/PRF/5 cells have low AKR1C3 expression (Fig. 3A). We established AKR1C3 knockdown Huh7 cells and AKR1C3 overexpressing cell lines in Hep3B and PLC/PRF/5 to examine its effect on cell growth (Fig. 3A). The results showed that AKR1C3 knockdown in Huh7 cells decreased cell growth, while AKR1C3 overexpression slightly enhanced Hep3B and PLC/PRF/5 cell growth *in vitro* (Fig. 3A). The colony formation assay showed that AKR1C3 knockdown in Huh7 cells reduced the colony numbers (Fig. 3C), although AKR1C3 overexpression in Hep3B cells had no effect (Suppl. Fig. S3). To confirm these results, we used the AKR1C3 inhibitor indomethacin [36,37]. Our analyses revealed that indomethacin effectively suppressed Huh7 cell growth and colony formation in a dose-dependent manner (Figs. 3B and 3D).

To examine whether AKR1C3 affected HCC growth *in vivo*, control and AKR1C3 knockdown Huh7 cells were injected subcutaneously into nude mice. As shown in Fig. 3E, the knockdown of AKR1C3 in Huh7 cells decreased cell growth *in vivo*, and the tumor weight was significantly lower than that of the vector control group. Taken together, these results demonstrate that AKR1C3 enhances the growth and colony formation ability of some HCC cell lines *in vitro* and *in vivo*.

### AKR1C3 expression is associated with changes in steroid hormone and prostaglandin metabolism in HCC tissues

To investigate the possible mechanism of AKR1C3-mediated regulation of HCC growth, we divided the 182 HCC samples from the GEO database (Fig. 2A) into two halves based on the AKR1C3 level and analyzed the differentially expressed genes. There were 223 differentially expressed genes (fold change ≥ 1.5, *p* < 0.05) in the AKR1C3-high group *vs*. the AKR1C3-low group (Fig. 4A). Pathway analysis showed that most of these significantly altered genes were involved in steroid hormone, prostaglandin, and arachidonic acid metabolism (Fig. 4B). We performed the same analysis for the TCGA HCC cohort, and 889 genes were differentially expressed in AKR1C3-high HCC tissues (Fig. 4C). Consistent with the results of GEO dataset analysis, the significantly changed genes were involved in steroid hormone, prostaglandin, and arachidonic acid metabolism (Fig. 4D).

AKR1C3 catalyzes the synthesis of high-affinity sex hormones and PGF2α. Many studies have explored the functions of sex hormones in HCC, but the role of PGF2α in HCC is unknown. PGF2α is upregulated in human endometrial adenocarcinoma, and PGF2α binding to PTGFR promotes the growth and production of PGE and PGF in tumor cells [20]. To study which signaling pathway is associated with AKR1C3, we analyzed the association between AKR1C3 levels and the downstream target genes of sex hormones or PGF2α using the TCGA dataset. The results revealed that AKR1C3 expression was positively correlated with the expression of two PTGFR downstream targets, *PTGES2* and *PTGES3* (Fig. 4E). In contrast, no correlation was found between AKR1C3 expression and the expression of the tested androgen or estrogen target genes, including *AR, PEG10, EZH2, NLRP3, TRIM25*, or *NOS3* (Suppl. Fig. S4). These analyses show that AKR1C3 upregulation is associated with increases in the expression of PTGFR downstream genes in human HCC tissues.

### AKR1C3 supports Huh7 cell growth by producing PGF2α and inhibition of PTGFR suppresses Huh7 cell growth

**Figure 5:**
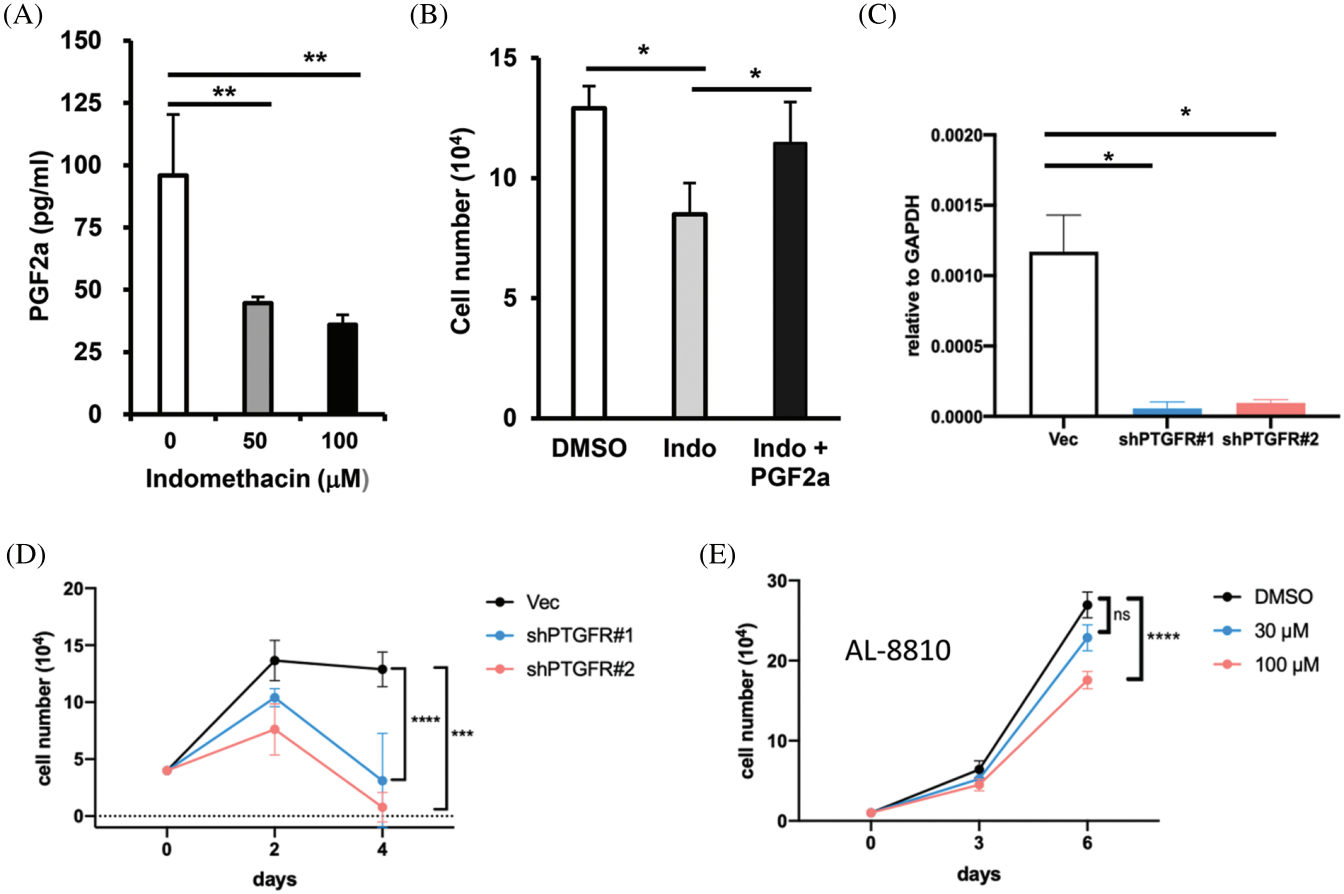
AKR1C3 supports HCC cell growth by producing PGF2α and suppression of PTGFR reduces HCC growth. (A) Suppression of AKR1C3 reduced the secretion of PGF2α in Huh7 cells. The PGF2α levels in the conditioned media from vehicle control- and indomethacin-treated Huh7 cells were measured using ELISA. The average PGF2α concentration (n = 4) is shown. (B) Exogenous PGF2α restored the growth of indomethacin-treated Huh7 cells. The cells were treated with DMSO control, 100 μM indomethacin, or indomethacin plus 2 μM PGF2α for 6 days. Representative results from 3 experiments are shown. (C) The expression of PTGFR in the vector control or two PTGFR knockdown stable lines was examined using RT-q-PCR. (D) Knockdown of PTGFR inhibited the growth of Huh7 cells. The average cell number from 3 experiments is shown. (E) The PTGFR antagonist AL-8810 suppressed Huh7 cell growth. The average cell number from 3 experiments is shown. *p < 0.05; **p < 0.01; ***p < 0.001; ****p < 0.0001.

Because AKR1C3 expression is associated with PTGFR downstream genes, we hypothesized that AKR1C3 regulates HCC growth by generating PGF2α. PGF2α was detected in the conditioned medium of Huh7 cells. When Huh7 cells were treated with the AKR1C3 inhibitor indomethacin, the PGF2α level was decreased (Fig. 5A). We studied whether the reduction in PGF2α was responsible for the growth suppressive effect of indomethacin. As shown in Fig. 5B, the addition of exogenous PGF2α restored the growth of indomethacin-treated Huh7 cells. These results indicate that AKR1C3 promotes Huh7 growth by producing PGF2α.

PGF2α has been reported to bind PTGFR and stimulate the proliferation of endometrial adenocarcinoma cells [20]. Because the role of PGF2α in HCC is unknown, we decided to assess whether PTGFR regulates Huh7 growth. We established two stable PTGFR knockdown lines (Fig. 5C) and examined their growth rate. We found that the knockdown of PTGFR significantly suppressed the growth of Huh7 cells *in vitro* (Fig. 5D). Furthermore, the PTGFR antagonist AL-8810 [38] suppressed Huh7 cell growth in a dose-dependent manner (Fig. 5E). These results show that in the AKR1C3-high expressing Huh7 cells, reducing PTGFR levels or antagonizing PTGFR impaired cell growth. Thus blocking the PGF2α-PTGFR axis may be a novel strategy to limit the growth of AKR1C3-positive HCC cells.

### Indomethacin increases the sensitivity of Huh7 cells to sorafenib

**Figure 6:**
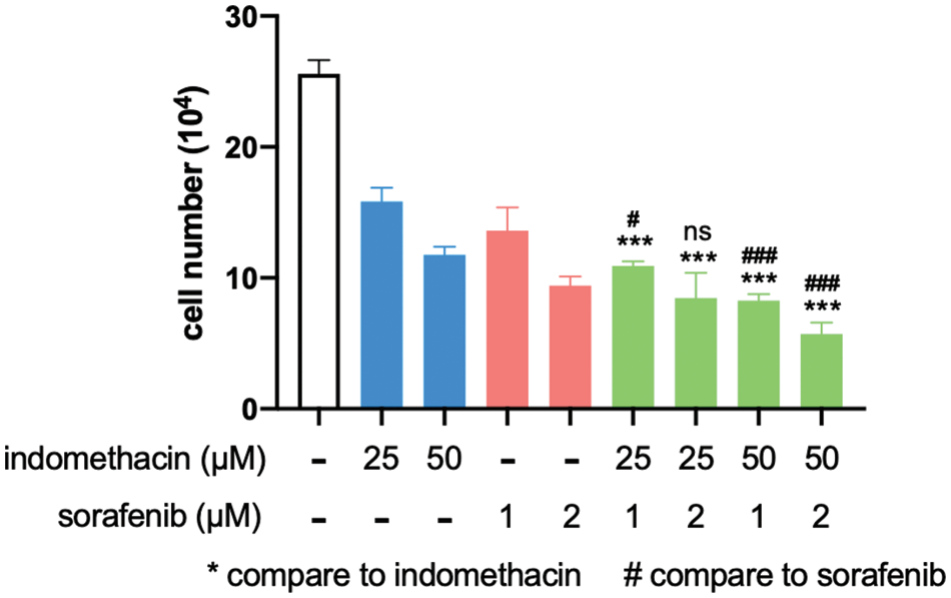
Indomethacin enhances the suppressive effect of sorafenib. Huh7 cells were treated with various concentrations of inhibitors, and the cells were counted after 6 days. The average cell number from 3 experiments is shown. ***p < 0.001.

For late-stage HCC patients, sorafenib is one of the very few drugs approved for chemotherapy. However, it only prolongs patient survival for three months and has a significant spectrum of side effects [4,39]. Because AKR1C3 is associated with doxorubicin resistance in breast cancer cells [15], we assessed whether suppression of its activity increases the sensitivity of HCC cells to sorafenib. As shown in Fig. 6, the addition of suboptimal doses of indomethacin decreased cell growth, and sorafenib had a similar effect. When we combined indomethacin with sorafenib, cell growth was further reduced compared to that in the sorafenib treatment alone group. Thus, the combination of an AKR1C3 inhibitor with sorafenib may enhance the anti-HCC effect of sorafenib.

## Discussion

AKR1C3 and its product PGF2α have been shown to control the development of prostate, breast, and endometrial carcinomas, respectively, but their role in HCC is largely unknown. Upregulation of AKR1C3 was detected in HCC tissues [16,17]. We confirmed that AKR1C3 expression was increased in human HCC tissues, and this phenotype was associated with a poor prognosis. Our results demonstrated that AKR1C3 promoted the growth of HCC cells *in vitro* and *in vivo*, and the growth-promoting activity was mediated by the production of PGF2α. Our findings indicate the possibility of blocking the AKR1C3-PGF-PTGFR axis in the treatment of HCC, which may improve the efficacy of current therapies.

The present study revealed that AKR1C3 promotes the proliferation and colony formation of some human HCC cells *in vitro* and that AKR1C3 is important for tumor growth in nude mice. Our observations are consistent with a recent study that reported a feed-forward loop of AKR1C3-NF-κB-STAT3 that facilitates the proliferation and metastasis of HCC cells. AKR1C3 upregulation enhanced NF-κB activation, which led to IL-6 production and subsequent HCC proliferation [17]. We analyzed gene expression profiles in the TCGA databases and identified two PTGFR downstream genes, *PTGES2* and *PTGES3*, that were associated with AKR1C3 levels in HCC. Therefore, we speculated that the production of PGF2α may play a role in the growth-promoting effect of AKR1C3. PGF-PTGFR autocrine loop has been shown to have various functions in the tumorigenesis of endometrial adenocarcinoma [20–22]. PGF2α also stimulates the migration and invasion of colorectal carcinoma cells and the survival of breast cancer cells [23,24]. However, the role of the PGF2α-PTGFR pathway in HCC is unknown. The results of the present study support that the production of PGF2α is another mechanism by which AKR1C3 enhances HCC growth. First, suppression of AKR1C3 leads to decreased secretion of PGF2α. Second, exogenous PGF2α restores the proliferation of indomethacin-treated Huh7 cells. Finally, the knockdown of PTGFR in Huh7 cells markedly suppresses cell growth. These results reveal the importance of the PGF2α-PTGFR axis in the growth of HCC cells. To the best of our knowledge, this is the first study to identify the role of the PGF2α-PTGFR pathway in HCC. This finding is particularly interesting because PGF2α is produced by only the uterus under normal physiological conditions. Thus it is worth further evaluating strategies for either blocking PGF2α generation or antagonizing PTGFR in the treatment of AKR1C3-positive HCC in the near future.

AKR1C3 also catalyzes the formation of high-affinity androgens and estrogens. In prostate cancer, AKR1C3 is upregulated by androgen-deprivation therapy, which confers resistance to androgen receptor (AR) antagonists, such as enzalutamide [13]. Mechanistically, AKR1C3 activates AR signaling by producing androgens and serving as a coactivator of AR [11,13,14]. Because the incidence of HCC is higher in males, sex hormones are assumed to be key factors in the development of liver cancer. Here we focused on the role of the PGF-PTGFR axis in AKR1C3-mediated cell growth because we only detected a positive correlation between AKR1C3 and PTGFR target genes. Given that estrogen suppresses HCC growth *in vitro* and *in vivo* [40,41], it is less likely to participate in the growth-promoting function of AKR1C3. On the other hand, we cannot exclude the possible contribution of androgens and AR to the function of AKR1C3. AKR1C3 promotes tumor growth in prostate cancer cells by increasing androgen production or activating AR-mediated signaling [11,13,14]. Although we found no correlation between AKR1C3 and AR or two androgen target genes, *PEG10* and *EZH2*, in the TCGA database (Fig. S3), more investigations are needed to clarify the role of the androgen/AR in the AKR1C3-promoting function in HCC.

Overexpression of AKR1C3 is detected in many cancer types and it contributes to tumorigenesis and drug resistance via various mechanisms [42]. As one of the AKR family members, AKR1C3 inactivates chemotherapeutic drugs by catalyzing carbonyl reduction, reducing quinones to hydroquinones, and inactivating 4-hydroxy-2-nonenal. Furthermore, AKR1C3 mediates doxorubicin resistance in breast cancer cells by decreasing PTEN expression and subsequently activating AKT [15]. Because AKR1C3 overexpression is associated with drug resistance in prostate and breast cancers [13,15], we hypothesized that suppression of AKR1C3 activity would increase the sensitivity of HCC cells to sorafenib. Our data showed that the combination of indomethacin and sorafenib enhanced the growth suppression in Huh7 cells (Fig. 6), indicating that high expression of AKR1C3 may contribute to the drug resistance of HCC. Consistent with this observation, a recent paper demonstrated that AKR1C3 was highly expressed in sorafenib-resistant HCC patients, and knockdown of AKR1C3 in HCC cell lines increased the sensitivity to sorafenib by inhibiting AKT activation [43]. Collectively, AKR1C3 may be a critical factor in the development of drug resistance in advanced HCC. The development and use of specific drugs targeting AKR1C3 may improve the response of end-stage HCC patients to chemotherapy.

### Limitations of this study

Using adjacent normal liver and HCC tissues collected in FEMH, we confirmed an increase in AKR1C3 mRNA and protein expression in the HCC tissues. Because the total patient number was only 21, we failed to find a correlation between AKR1C3 expression and HCC patient survival. In addition, we did not check the PGF2α levels in the AKR1C3-high and AKR1C3-low HCC tissues. Analyzing the correlation between AKR1C3 and PGF2α levels in HCC tissues will help us clarify the role of the AKR1C3-PGF2α-PTGFR axis in HCC. Therefore, it is very important to investigate these questions in the near future. Due to a lack of good antibodies for detecting PTGFR, we failed to show the PTGFR protein levels in the knockdown Huh7 cell lines (Fig. 5). Confirmation of the knockdown effect would further clarify the role of PTGFR in the growth of HCC cells.

## Conclusion

In summary, we confirmed that AKR1C3 is upregulated in human HCC tissues and that this upregulation is associated with a poor prognosis in patients. We identified that the growth-promoting effect of AKR1C3 in HCC cells was mediated by generating PGF2α. Knockdown of PTGFR suppressed the growth of HCC cells. The AKR1C3 inhibitor indomethacin increased the sensitivity of HCC cells to sorafenib. Our findings indicate the possibility that targeting the AKR1C3-PGF2α-PTGFR axis may help improve the efficacy of HCC therapies.

## Supplementary Materials



Fig. S1 AKR1C3 expression was negatively associated with progression-free survival in the HCC patients from the TCGA database. A cohort of 365 patients with HCC in the TCGA database was used to analyze the correlation between AKR1C3 expression with progression-free survival. The medium expression level of AKR1C3 in the cohort was 70.34, and it is used to divide these patients into AKR1C3-high (n = 182) and AKR1C3-low groups (n = 183), and the progression-free survival rate was analyzed using the log-rank test.

Fig. S2 The expression of AKR1C3 mRNA was not correlated with tumor stage, race, age, weight, or risk factors in patients with HCC. After classifying patients in the TCGA liver cancer database according to different parameters, we analyzed the correlation between different factors and AKR1C3 expression. Because some clinical characteristics are not included in the HCC patients from the TCGA database, the sample size for each analysis is different. The patient number (n) in different groups was shown.

Fig. S3 AKR1C3 overexpression did not affect the colony formation of Hep3B cells. After confirming the protein levels using Western blot analysis, five thousand vector control or AKR1C3 overexpressing Hep3B cells were seeded on a 6-well plate, and the medium was changed every 3 days. After 14 days, the cells were washed, fixed, and stained with crystal violet. The colony number was evaluated using ImageJ, and the average colony number out of 3 independent experiments is shown.

Fig. S4 The expression of AKR1C3 is not associated with androgen or estrogen target genes in the HCC samples from the TCGA database. The correlation between AKR1C3 expression with the expression of each one of the androgen target genes (AR, PEG10, and EZH2) or estrogen target genes (NLRP3, TRIM25, and NOS3) from the TCGA database was analyzed using Spearman correlation.

## Data Availability

All the data and materials generated for this study are available on request to the corresponding author.

## Supplementary Materials

The supplementary material is available online at https://doi.org/10.32604/or.2023.030975.
