# Supplementary material for: Aldo-keto reductase family member C3 (AKR1C3) promotes hepatocellular carcinoma cell growth by producing prostaglandin F2α
Source: Oncol Res. 2023 Nov 15;32(1):163–74. doi: 10.32604/or.2023.030975 (PMC10767238; doi:10.32604/or.2023.030975)
Supplement: Supplementary file 1 [file OncolRes-32-30975-s001.docx]

| Table S1. Clinical characteristics of the 21 HCC carcinoma patients from Far Eastern Memorial Hospital | | |  |
| --- | --- | --- | --- |
| Age |  | 60^a^ | (43-80) |
| Male, n (%) |  | 13 (61.9%) |  |
| Tumor sizes (cm) |  | 4.7^a^ | (2.0-14.7) |
| Cirrhosis, n (%) |  | 14 (66.7%) |  |
| Child-Pugh classification, n (%) | A | 19 (90.5%) |  |
|  | B | 2 (9.5%) |  |
| Type, n (%) | HBV | 10 (47.6%) |  |
|  | HCV | 1 (4.8%) |  |
|  | HBV/Alc | 2 (9.5%) |  |
|  | non-HBV, non-HCV | 8 (38.1%) |  |
| AFP | Normal <9 ng/mL | 7 (36.8%) |  |
|  | Abnormal ≥ 9 ng/mL | 14 (66.7%) |  |
| Presence of satellite tumors, n (%) |  | 6 (28.6%) |  |
| TNM Stage, n (%) | I/II | 16 (76.2%) |  |
|  | III/IV | 5 (23.8%) |  |
| Grade of differentiation, n (%) | I/II | 13 (61.9%) |  |
|  | III | 8 (38.1%) |  |
| Vascular invasion, n (%) |  | 15 (71.4%) |  |
| Recurrence, n (%) |  | 7 (33.3%) |  |
| Survial, n (%) |  | 8 (38.1%) |  |
|  |  |  |  |
| Notes: |  |  |  |
| ^a^Data is represented as mean (range: lowest-highest). | |  |  |
| Abbreviations: HBV, hepatitis B viral infection; HCV, hepatitis C viral infection; Alc, alcoholism; AFP, alpha fetal protein. | | | |
| The normal AFP level is below 9 ng/mL |  |  |  |
| TNM stage: according to the 8th edition of AJCC | |  |  |
