# Supplementary figures and images for: Aldo-keto reductase family member C3 (AKR1C3) promotes hepatocellular carcinoma cell growth by producing prostaglandin F2α
Source: Oncol Res. 2023 Nov 15;32(1):163–74. doi: 10.32604/or.2023.030975 (PMC10767238; doi:10.32604/or.2023.030975)

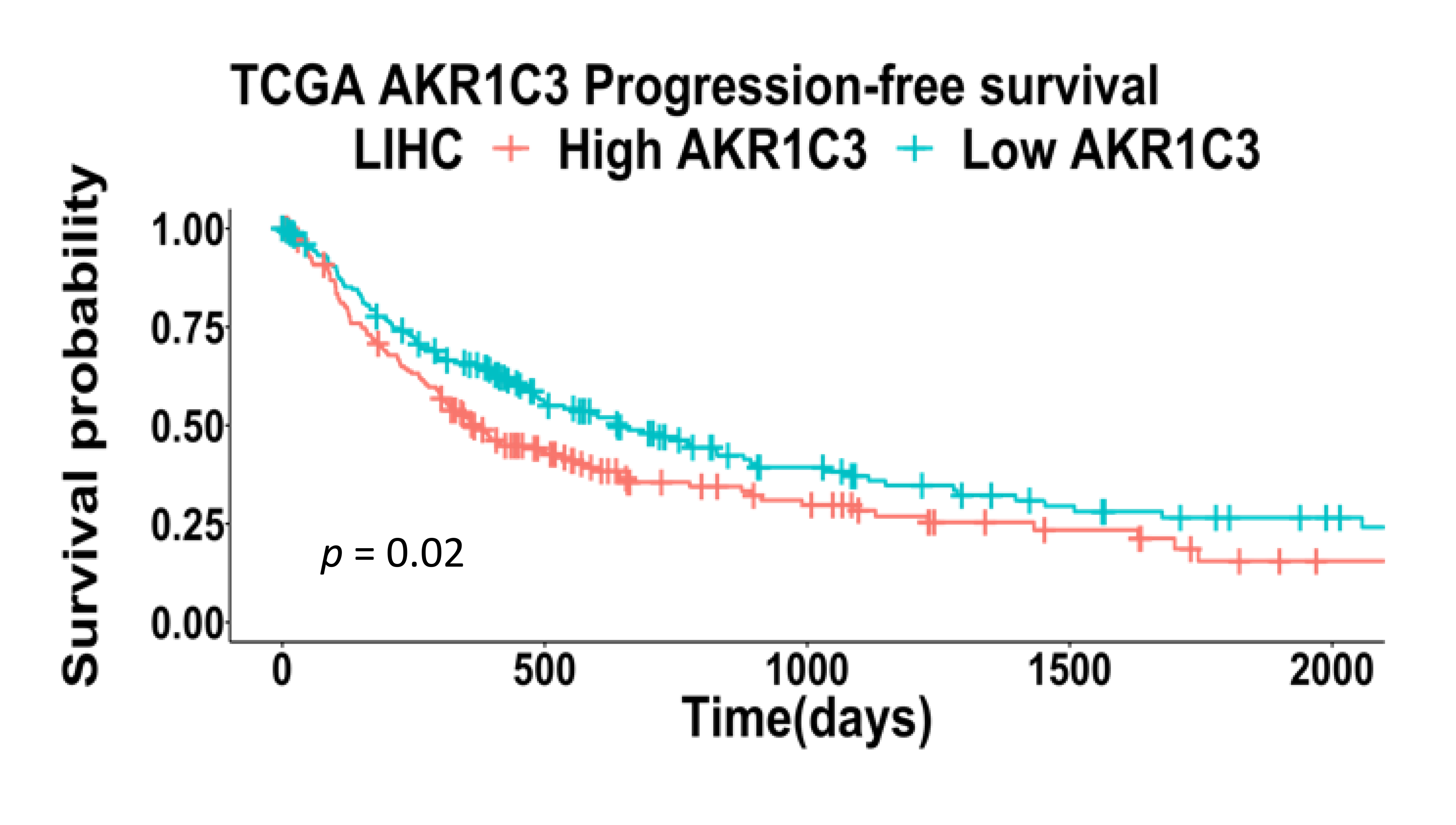

Supplement: Fig. S1 [file OncolRes-32-30975-s001.tif]

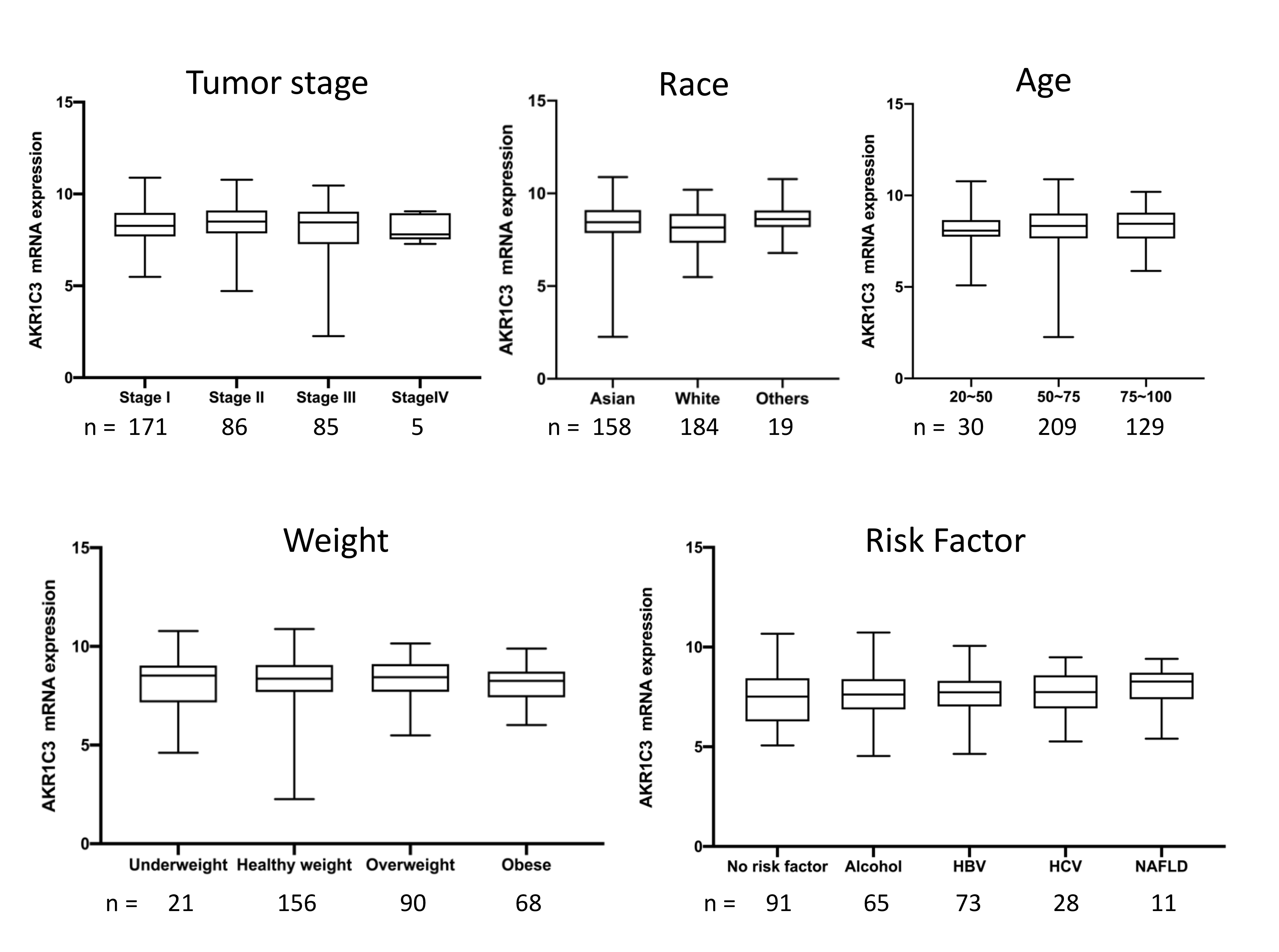

Supplement: Fig. S2 [file OncolRes-32-30975-s002.tif]

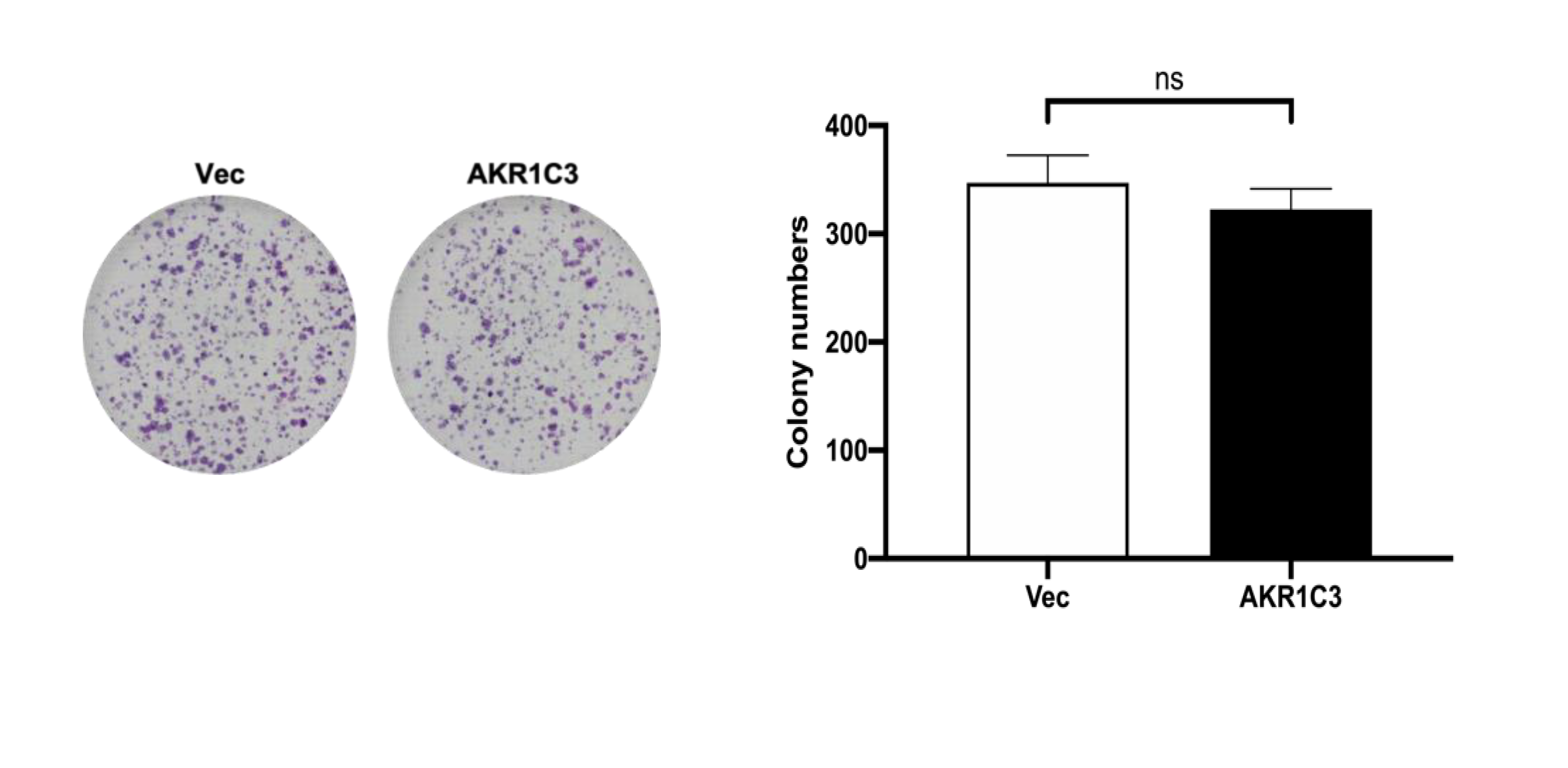

Supplement: Fig. S3 [file OncolRes-32-30975-s003.tif]

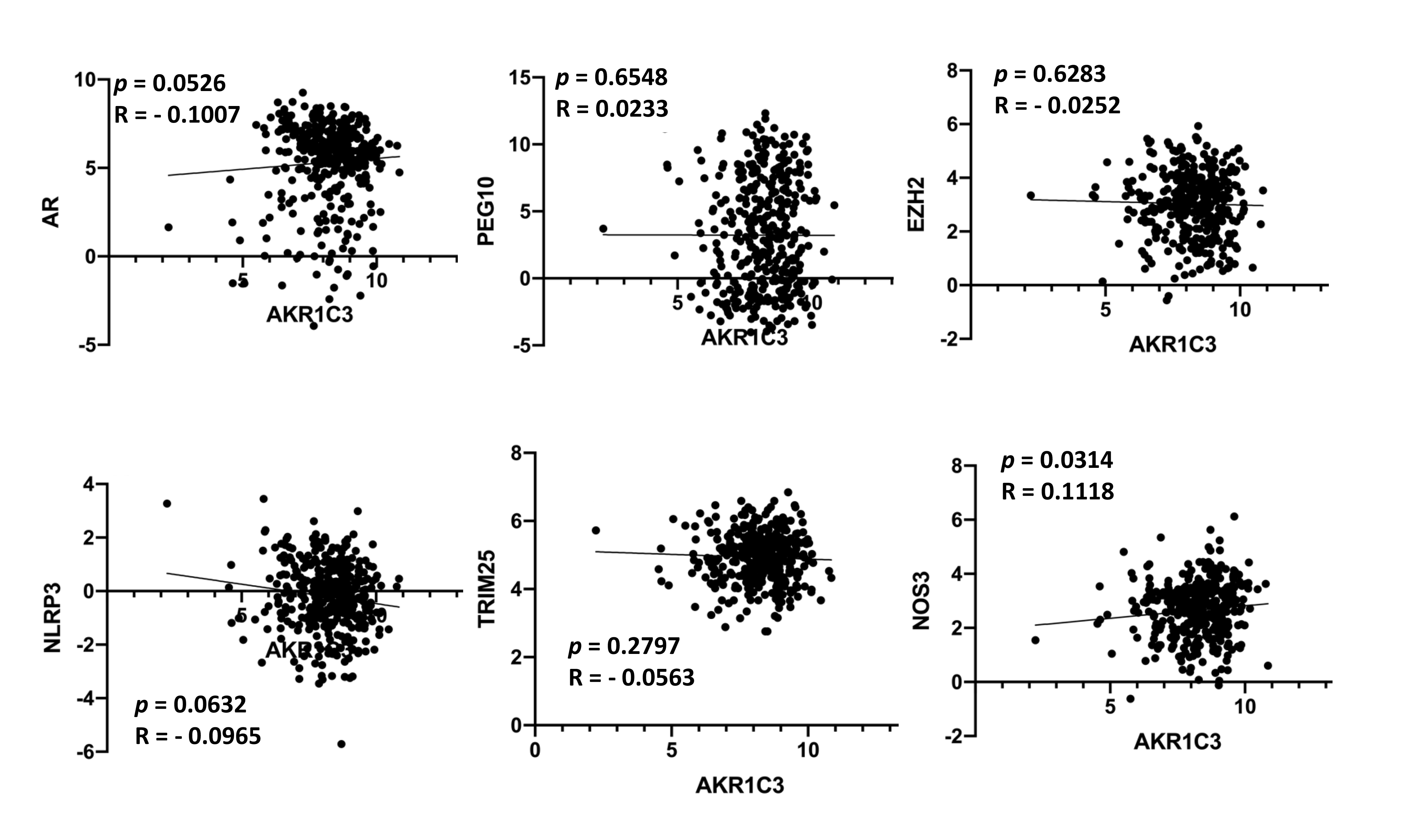

Supplement: Fig. S4 [file OncolRes-32-30975-s004.tif]
